# Pet Face: Mechanisms Underlying Human-Animal Relationships

**DOI:** 10.3389/fpsyg.2016.00298

**Published:** 2016-03-08

**Authors:** Marta Borgi, Francesca Cirulli

**Affiliations:** Section of Behavioral Neuroscience, Department of Cell Biology and Neurosciences, Istituto Superiore di SanitàRome, Italy

**Keywords:** attachment, baby schema, cuteness, dog, faces, oxytocin, pet animals

## Abstract

Accumulating behavioral and neurophysiological studies support the idea of infantile (cute) faces as highly biologically relevant stimuli rapidly and unconsciously capturing attention and eliciting positive/affectionate behaviors, including willingness to care. It has been hypothesized that the presence of infantile physical and behavioral features in companion (or pet) animals (i.e., dogs and cats) might form the basis of our attraction to these species. Preliminary evidence has indeed shown that the human attentional bias toward the baby schema may extend to animal facial configurations. In this review, the role of facial cues, specifically of infantile traits and facial signals (i.e., eyes gaze) as emotional and communicative signals is highlighted and discussed as regulating the human-animal bond, similarly to what can be observed in the adult-infant interaction context. Particular emphasis is given to the neuroendocrine regulation of the social bond between humans and animals through oxytocin secretion. Instead of considering companion animals as mere baby substitutes for their owners, in this review we highlight the central role of cats and dogs in human lives. Specifically, we consider the ability of companion animals to bond with humans as fulfilling the need for attention and emotional intimacy, thus serving similar psychological and adaptive functions as human-human friendships. In this context, facial cuteness is viewed not just as a releaser of care/parental behavior, but, more in general, as a trait motivating social engagement. To conclude, the impact of this information for applied disciplines is briefly described, particularly in consideration of the increasing evidence of the beneficial effects of contacts with animals for human health and wellbeing.

## Introduction

In the context of Wilson's ***Biophilia Hypothesis***(1984), the human need and propensity to focus on and to affiliate with animals (“Biophilia”), as well as its counterpart (negative attitudes toward some animals, or “Biophobia”), have been depicted as biological tendencies (Wilson, 1984; Kellert, 1993a). As shown by a number of experimental studies, a general proneness toward animals and animal stimuli seems to emerge from early childhood onward (DeLoache et al., 2011; Lobue et al., 2013; Muszkat et al., 2015). Even in subjects with a deficit in the social domain (i.e., children with autism spectrum disorders) preference for animal features has been shown (Celani, 2002; Prothmann et al., 2009, but see Grandgeorge et al., 2015), as well as an increase in social behaviors in the presence of animals compared to toys (O'Haire et al., 2013).

KEY CONCEPT 1 **Biophilia hypothesis**

The concept of Biophilia refers to a hypothetical human affinity for the living world. It can also refer to human tendency to interact and form close association and emotional bond with the other forms of life in nature. Based on this theory, the Biophilia is considered to be innate and felt universally by humans.

Why animals constitute such an attractive stimulus for humans has not been completely clarified. Living beings engage the attention of people more than objects do, and it has been hypothesized that the evolutionary reason behind this response is that paying attention to other living beings is significant for individual fitness (New et al., 2007; Mormann et al., 2011).

However, while research efforts have been dedicated to empirically confirm human “biophilic” (and/or “biophobic”) predisposition and its emergence during development, very little attention has been paid to the identification of specific animal attributes underpinning distinct behavioral responses in humans, particularly in children.

In this review, we will first report evidence of the effect of some animal physical traits on human perception of—and attitudes toward—different species. The specific role of animal facial cues as emotional and communicative signals regulating the human-animal bond will be then emphasized. In particular, infantile facial traits will be highlighted as a class of stimuli with high biological relevance, rapidly and unconsciously capturing attention and eliciting affectionate responses, including readiness to care and social engagement. In our contribution to the endeavor of better understanding the mechanisms underlying human attraction to animals, we demonstrated an early emergence of the response to infantile facial traits and its generalization to companion (pet) animals (i.e., dogs and cats; Borgi et al., 2014). Here this evidence will be viewed also in the light of recent findings showing the primary role of facial cues in regulating human-dog bond through oxytocin secretion (Nagasawa et al., 2015). To conclude, the impact of this information for human health, as well as for animal welfare and management, will be briefly described.

## [Animal] beauty is in the eye of the beholder

The study of human **attitudes** toward animals is an extremely complex issue, involving a multitude of evolutionary, psychological, and cultural aspects (Serpell, 2004). However, even not considering this variance, people's proneness toward, and consideration of, animal species greatly depend on some attributes intrinsic to the animal itself: both physical and behavioral characteristics of the various species largely influence human perception of animals and may explain why people like some animals, while disliking others (Serpell, 2004).

KEY CONCEPT 2 **Attitude**

An attitude can be defined as a feeling or opinion about a particular entity that is expressed by evaluating it with some degree of favor or disfavor, as well as a manner of thinking, feeling, or behaving that reflects this disposition.

A substantial body of literature on human attitudes and likeness of some species has shown that animals phylogenetically close to humans, and/or that are physically, behaviorally, or cognitively similar to them, tend to be preferred, evoke more positive affect, as well as higher concern in terms of welfare and conservation (Plous, 1993; Gunnthorsdottir, 2001; Tisdell et al., 2006; Martín-López et al., 2007; Knight, 2008; Batt, 2009). By contrast, humans show negative attitudes toward animals considered phylogenetically distant or dissimilar (e.g., reptiles, fishes, invertebrates, Kellert, 1993b; Bjerke et al., 1998; Bjerke and Ostdahl, 2004; Prokop et al., 2010).

**Figure 1:**
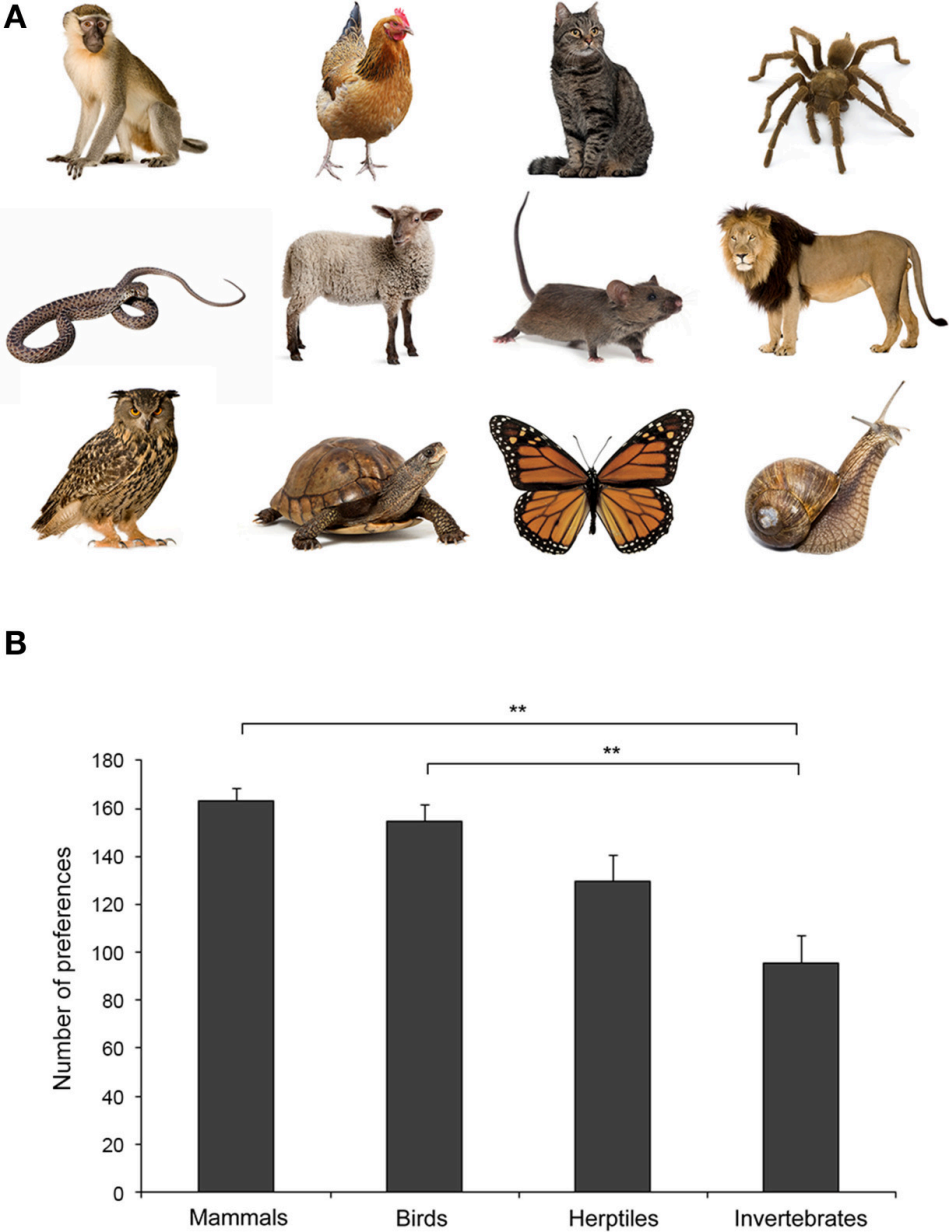
**(A)** Examples of stimuli presented to children. From the top (left): vervet monkey, chicken, cat, spider, rattlesnake, sheep, mouse, lion, eagle owl, turtle, monarch butterfly, and snail. Photos: Thinckstock/GettyImages. **(B)** Children's preference for different taxa (mean + SEM). A statistically significant difference between Mammals and Invertebrates and between Birds and Invertebrates is shown (^**^p < 0.01). For further details see Borgi and Cirulli (2015).

Borgi and Cirulli's analyses of kindergarten children's preferences for a wide range of different animal species are in line with the “similarity principle” (Tisdell et al., 2006) and suggest an early emergence of such a predisposition (Borgi and Cirulli, 2015; Figure 1).

However, similarity to humans, in particular phylogenetic closeness, is only one of the animal attributes explaining the enormous variance in people's attitudes toward animals (Serpell, 2004). Animal physical appearance, including aesthetic qualities (e.g., color; Stokes, 2007; Lišková and Frynta, 2013), was shown to be a salient factor underlying human attitudes toward animals. Anthropomorphic features, large size and neotenous (juvenile) traits, represent the animal attributes that have been most consistently shown to affect human preferences and attitudes (Serpell, 2004) and that contribute to preference's forming (Borgi and Cirulli, 2013).

## Why do we like animals? baby schema and cute response

In the literature it is claimed that humans tend to prefer animals that they perceive as aesthetically appealing or “cute” (Gould, 1979; Woods, 2000; Gunnthorsdottir, 2001; Knight, 2008; Archer and Monton, 2011; Herzog, 2011b). Cuteness is often used as a measure indicative of attractiveness to a stimulus commonly associated with infancy and youth. The term was conceptualized in the Konrad Lorenz's notion of *Kindchenschema* (or **baby schema**) and first described by the ethologist as a set of facial features (i.e., large head and a round face, a high and protruding forehead, large eyes, and a small nose and mouth) able to trigger an innate releasing mechanism for caregiving and affective orientation toward infants (Lorenz, 1943). More recently, several empirical studies have shown that faces with these traits are commonly perceived as cute and attractive and are consistently preferred to those with a less infantile facial configuration (Sanefuji et al., 2007; Glocker et al., 2009a; Lobmaier et al., 2010; Luo et al., 2011; Little, 2012).

KEY CONCEPT 3 **Baby schema**

The baby schema (Kindchenschema), as proposed by ethologist Konrad Lorenz, is a set of infantile physical features perceived as cute and motivates caretaking behavior in other individuals, therefore providing the fundamental function of enhancing offspring survival.

The concept of cuteness not only encompasses the processing of specific morphological features, but also involves a positive/affectionate behavioral response. Increased attention and willingness to care, positive affect and protective behavior, as well as decreased likelihood of aggression toward the infant, characterize the so-called *baby schema response* or *cute response* (Alley, 1983; Brosch et al., 2007; Sherman et al., 2009; Glocker et al., 2009a; Nittono et al., 2012). In species whose young completely depend on their caregivers for sustenance and protection, such response has a clear adaptive value, contributing to enhance offspring chances of survival (Lorenz, 1943) and helping mothers to focus on newborns and modulating attachment (Sprengelmeyer et al., 2009).

The idea that the human response to infantile features is not restricted to conspecifics, but can also be elicited by heterospecifics was first proposed by Lorenz and was subsequently demonstrated by several empirical studies which have shown the generalization of the cute response to real animals (Sherman et al., 2009; Archer and Monton, 2011; Little, 2012; Golle et al., 2013; Lehmann et al., 2013), representations of animals such as cartoon characters (e.g., Mickey Mouse, Gould, 1979) and stuffed/toy animals (e.g., Teddy bear, Hinde and Barden, 1985; Archer and Monton, 2011).

**Figure 2:**
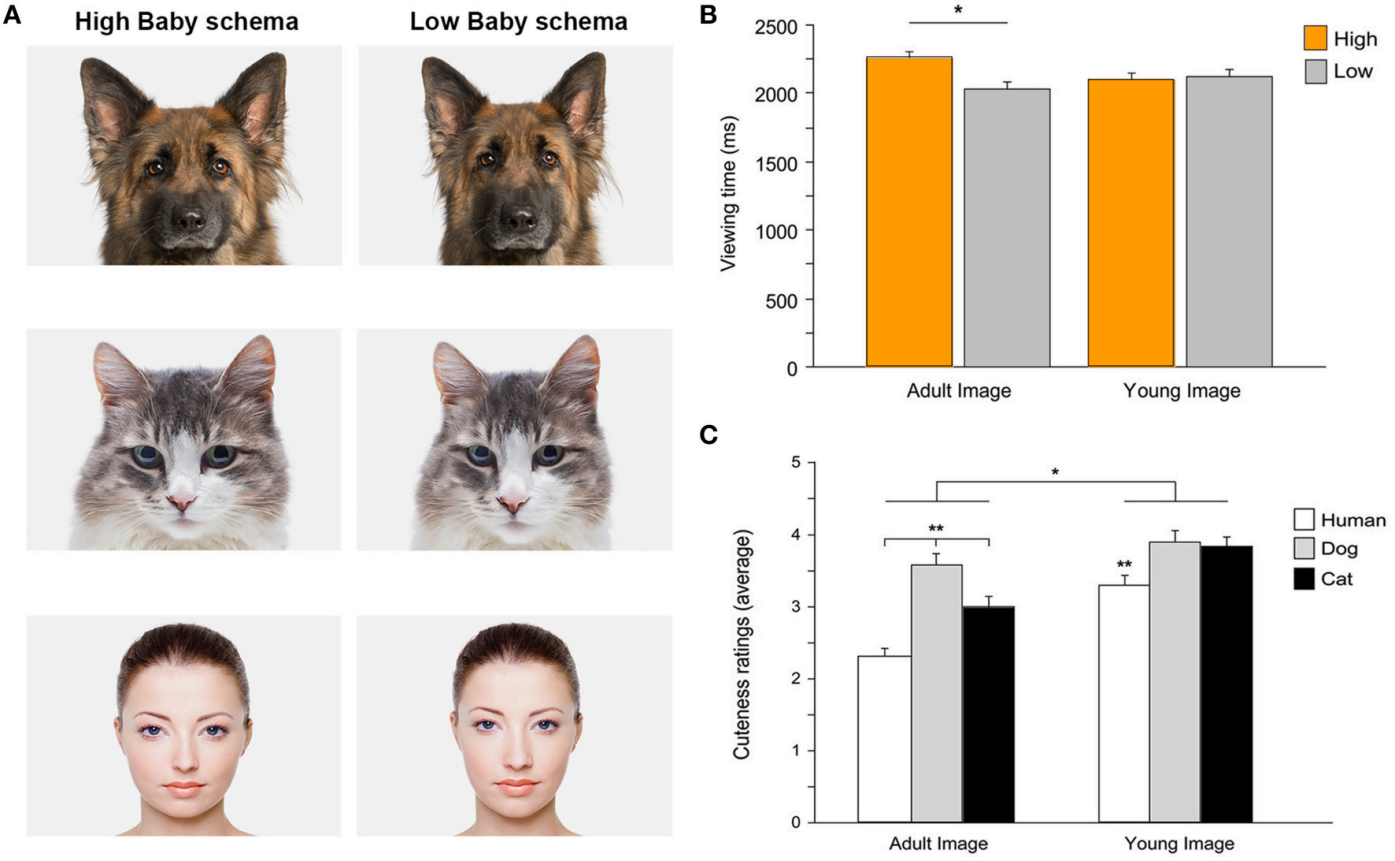
**(A)** Examples of stimuli presented to participants. High infantile version (on the left) and low infantile version (on the right) of dog, cat, and human faces. Photos: Thinkstock/Getty Images (modified). **(B)** Children's preferential looking. Viewing time directed to high and low versions of images depicting adult and young faces (mean + SEM). Attentional bias for high infantile faces was evident when children viewed adult images (^*^p < 0.05), while no difference was found when viewing images of young faces. **(C)** Cuteness ratings. Average cuteness ratings given to images of adult and young faces of three species (human, dog, and cat) (mean + SEM). Overall, faces of young individuals were rated as cuter than those of adults (^*^p < 0.05). Some species-specific effects were also shown (^**^p < 0.01). Adapted from Borgi et al. (2014).

Analyses on the emergence of a cute response during development and its extension to the human-animal context are scarce (Fullard and Reiling, 1976; Maestripieri and Pelka, 2002; Sanefuji et al., 2007). In a recent study Borgi et al. (2014) have investigated the effects of the baby schema on children's perception of cuteness in human and animal faces, using both explicit (i.e., cuteness judgment) and implicit (i.e., gaze behavior) measures. In this study, the effect of the baby schema on cuteness perception and attentional response was assessed in young children (3–6 years old) using eye-tracking techniques and a controlled design in which stimuli (human, dog, and cat faces) were objectively quantified according to the baby schema content. Overall this study has shown that the response to an infantile facial configuration emerges early during development. The manipulation of infant-like traits affected both cuteness perception and gaze allocation to infantile stimuli and to specific facial features (i.e., the eyes), an effect not simply limited to human faces (Borgi et al., 2014; Figure 2).

### Neural systems underlying attraction to infant faces

Accumulating behavioral and neurophysiological studies support the idea of infantile faces as highly biologically relevant stimuli rapidly and unconsciously capturing attention and eliciting positive emotions (Brosch et al., 2007; Kringelbach et al., 2008; Caria et al., 2012; Senese et al., 2013; Borgi et al., 2014; Esposito et al., 2014). Adults' automatic emotional responses to infant stimuli appear to be mediated predominantly by the autonomic nervous system, independently of caregiving experience, or cultural exposure (Esposito et al., 2014). The evidence indicates an extensive neural circuitry involved in the perception of infant faces: enhanced activation has been found in brain areas involved in face perception, attention, reward and emotion processing, empathy, and motor control (for a review see Luo et al., 2015).

Overall, consistent behavioral and neurophysiological findings suggest that “*the structural configuration of infant faces might act as a heightened attentional/emotional biasing mechanism*” (Kringelbach et al., 2008). Moreover, the observed activation of specific brain circuits involved in preparation of voluntary action in response to infant faces (Caria et al., 2012), as well as the improved performance on fine motor dexterity tasks that required carefulness after viewing cute images (Nittono et al., 2012), were interpreted as reflecting a “readiness” to interact with babies, as well as preparation and intention to communicate.

Studies on non-parents indicate a generalized inclination to respond positively even to unfamiliar infant faces. In particular, the activation of reward circuits previously shown to mediate attachment and caregiving behaviors in parents toward their own children (i.e., dopamine-associated reward-processing areas, Swain et al., 2007) confirms the notion of infant faces as rewarding and salient stimuli trascending the biological relationships (Kringelbach et al., 2008; Caria et al., 2012).

The extent to which the brain circuits underlying essential adults' responsiveness to human infants also subserve a general inclination to respond to infant animal stimuli is still unclear. Some evidence exists indicating a species-specific adult brain response, i.e., a disposition to respond specifically to human children, instead of a more general inclination toward infant stimuli. In particular, in Caria et al. (2012) brain circuits involved in adults' preparation for communicative behavior with infants were activated preferentially by human infant faces, when compared to animal faces. A perception bias to conspecifics was also shown in another study (Brosch et al., 2007), that is an increase in spatial deployment of attentional resources in response to human infant faces compared to dog and cat faces.

It should be taken into account that most previous studies have used facial stimuli not objectively quantified in term of baby schema content. Hence the interpretation of outcomes is limited by the difficulty to dissociate the response to a specific stimulus (i.e., humans vs. animals; adult vs. young) from the response to its facial configuration (i.e., baby schema). By developing an effective procedure to create stimuli with parametrically manipulated baby schema content—and that retained all the characteristic of the individual portrait—Glocker and colleagues have shown that images with more pronounced baby schema elicit stronger motivation for caregiving compared with low baby schema images (Glocker et al., 2009a), also suggesting a neurophysiologic mechanism by which the baby schema could promote human nurturing behavior (Glocker et al., 2009b). Images with a higher baby schema content were shown to activate brain regions such as the nucleus accumbens, a key structure of the mesocorticolimbic system involved in the anticipation of reward, suggesting the role of infantile facial traits in providing a motivational drive to caretaking behavior (Glocker et al., 2009b). By applying the same procedure to modify dog and cat faces, Borgi et al. (2014) showed that the human attentional bias toward the baby schema may extend to animal facial configurations. This generalized pattern—and its extension beyond the mother-infant relationship context—speaks to the efficacy of baby-like appearances in eliciting alloparental care and may explain why we feel the urge to hold and care for anything that resembles a baby.

## Pets as baby substitutes?

It is well known that some animals, such as the most common pet species (i.e., dogs and cats), exhibit lifelong morphological and behavioral infantile characteristics. The retention of youthful traits into adulthood (i.e., *neoteny*) is considered a by-product of the domestication process (Belyaev, 1979; Frank and Frank, 1982; Hare et al., 2005), which may have operated through generations of conscious or unconscious selective breeding for non-aggressive behavior toward humans (i.e., tameness or docility, Belyaev, 1979). In comparison with their progenitors, domestic dogs are smaller in size, have shortened facial region and exhibit behaviors typical of young wolves (e.g., barking, whining, soliciting attention) throughout their lifetime (Morey, 1994). Infantile characteristics have been particularly emphasized during human selection of certain breeds for aesthetic reasons, often with negative consequences in terms of animal welfare (Serpell, 2002; King et al., 2012).

It has been hypothesized that both behavioral and physical infantile features present in companion animals might form the basis of our attraction to these animals and may bear some part of the responsibility for our motivational drive to pet-keeping and pet-caretaking (Archer, 1997).

We won't give here a full account of the variety of observable facts of animals treated like human infants (e.g., toy or “handbag” dogs dressed as babies), a reality which appears to be part of the more complex phenomenon known as anthropomorphism (i.e., the attribution of human characteristics or behavior to any other non-human entity in the environment, Urquiza-Haas and Kotrschal, 2015), and thus beyond the scope of this review. Instead, what is of particular interest, especially from a research perspective, is the undeniable fact that the bond between owners and their companion animals shares remarkable similarities to the relationship between human parents (typically the mother) and their children. These similarities have been described within the framework of human **attachment theory** (Bowlby, 1969; Ainsworth and Bowlby, 1991), whose patterns of relationships may also be applied to the formation and maintenance of people's bond with their companion animals. Shared features of the two relationships include: dependency, proximity seeking, caregiving, and feelings of affection, which ultimately ensure security, comfort, protection, and survival to the child, as well as to the dog (Topál et al., 2005; Payne et al., 2015).

KEY CONCEPT 4 **Attachment theory**

As proposed by the psychiatrist John Bowlby, humans possess a primary motivational drive to form close affectional bonds. During childhood, a secure attachment to the caregiver (often but not necessarily the mother) provides a secure base from which an infant can explore the environment and on which he/she can form lasting, secure, and intimate bonds during adulthood.

The presence of a human being can attenuate the effect of a stressful event in dogs (known as safe haven effect, Gacsi et al., 2013) which appear to use the owner as a secure base for interacting with the environment (Horn et al., 2013). Moreover, previous studies have shown that the language used to talk to animals mimics the so-called *motherese* or *baby talk* (Burnham et al., 2002). Whether an association exists between the strength of the relationship an owner feels to his/her dog and the dog's attachment profile to its owner is still disputed (Siniscalchi et al., 2013).

### Neurophysiological correlates of the human-animal bond

Notwithstanding human-pet relationships are considered an interspecific form of attachment, the growing body of research on the neurophysiological basis of attachment and caregiving systems—and the interaction between them (Lenzi et al., 2015)—have almost completely neglected the analysis of the human-pet bond. Only very recently Stoeckel et al. (2014) have reported a comparison of fMRI-related brain activation patterns in women viewing facial images of their own child and own dog. Results show regions implicated in emotion, reward, and affiliation, as well as memory, visual/face processing, and social cognition, all showing increased activity when participants viewed either their own child or their own dog (Stoeckel et al., 2014). However, images of their own child, but not of their own dog, activated additional regions involved in reward function and known to have a critical function for human-human relationships of evolutionary importance (i.e., romantic relationships and mother-infant bonding, Bartels and Zeki, 2004). By contrast, greater magnitude and extent of activation in regions central to visual/face processing and social cognition was elicited in response to own dog images compared to own child images (Stoeckel et al., 2014). This evidence was interpreted by the authors as reflecting the more central role of facial signals (i.e., facial expressions and gaze), in dog-human interactions, compared to human-human communication, mainly due to the absence of verbal language.

Consistent with these considerations, the primary role of facial cues (namely the mutual gaze) as emotional and communicative signals during interactions between owners and their dogs was recently highlighted and interpreted as regulating the human-dog bond, similarly to what was observed in the adult-infant interaction context (Nagasawa et al., 2009, 2015). Mutual gaze in infant-caregiver dyads is considered an attachment behavior (De Dreu et al., 2010) and a marker of social engagement, with its primary role in regulating social bonding, mainly through maternal oxytocin secretion (Farran and Kasari, 1990; Feldman et al., 2007; Nagasawa et al., 2012). Oxytocin is implicated in the **neuroendocrine regulation** of maternal behavior (Rilling and Young, 2014) and oxytocin signaling appears to be critical in social bond formation (Hurlemann and Scheele, 2016; Numan and Young, 2016). In a recent study based at Azabu University in Japan, Miho Nagasawa and colleagues have shown an association between dog's gaze and urinary oxytocin concentrations in their owners during affiliative interactions; the raise in oxytocin facilitated owners' affiliation toward their dogs with a consequent increase in oxytocin concentration also in the animal (Nagasawa et al., 2015). The failure to demonstrate such “interspecies oxytocin-mediated positive loop” in human-(hand-raised)wolf dyads suggests the acquisition of human-like communication modes during dog's domestication, mainly as a result of a selection on systems mediating fear and aggression toward humans (the so-called “emotional evolution,” Hare and Tomasello, 2005; Nagasawa et al., 2015). The evidence provided by the Nagasawa's team not only suggests that “*dogs were domesticated by coopting social cognitive systems in humans that are involved in social attachment”* (Nagasawa et al., 2015), but also shows how this acquisition may contribute to the establishment of a human-animal bond that presents both behavioral and neurohormonal similarities with the mother-infant relationship.

KEY CONCEPT 5 **Neuroendocrine regulation**

The concept of neuroendocrine regulation comprises the role of hormones in regulating motivational states. Steroids and peptides, by influencing central states, provide the motivational drive for sexual or parental behaviors, feeding, just to name a few. This notion also encompasses the ability of physiological systems to respond to social and physical stimuli.

### The “F-word”: pets as friends

Scientific evidence reviewed so far points in the direction of a similarity between human-pet and human-infant relationship and suggests the role of facial traits, namely the baby schema, in modulating the release of human care/“parental” behavior toward domesticated species (Lorenz, 1943). Sherman and Haidt's argument contradicts this notion and considers the baby schema response as an emotional response “releasing” social behaviors, such as play and other affiliative interactions, and only indirectly leading to caregiving (Sherman and Haidt, 2011). In this context, authors rethink cuteness as not simply triggering care/parental behavior, but more in general as motivating social engagement. This vision would explain a variety of evidence on the existence of the baby schema response beyond the parent-infant relationship, e.g., responsiveness toward non-kin children and animals, use of infantile traits in toys, cartoons, and robots (Sherman and Haidt, 2011). This would also explain the common tendency to anthropomorphize cute objects and animals, in this context proposed as a mechanism to achieve social connection with them (*sociality motivation*, Serpell, 2002; Epley et al., 2008).

The farm fox experiment conducted in Siberia on silver foxes proved that selecting for a “friendly” behavior can neotenize adult temperament and morphology, by altering the genes controlling systems—such as the HPA axis—modulating both fear and aggression (Belyaev, 1979; Trut, 1999; Hare et al., 2005; Trut et al., 2009). Craniofacial proportions that we find attractive and (cute) in conspecific and animal faces might therefore be considered as a sign of a friendly predisposition to interact and as genetically and hormonally linked to the evolution of social contact, trust and, ultimately, cooperation (Elia, 2013). As a matter of fact, baby-faced adults are considered more warm, likeable and friendly than less cute individuals (Zebrowitz and Montepare, 1982; McArthur and Apatow, 1983; Berry, 1991).

As in Elia (2013), “friendly” here refers to “calm, eager-to-interact individuals” and thus comprises the behavioral bases of **friendship**. The question is whether dogs, “man's best friends,” and other pet species, could be actually considered as friends.

KEY CONCEPT 6 **Friendship**

The term friendship can be used interchangeably with the term social bond; however, the first is often considered a hallmark of humans. Friendship can be defined on the basis of the patterning and quality of interactions, that is, between friends the frequency and consistency of affiliative interactions is greater than between non-friends and lasts longer.

More than a decade has passed since the seminal work of J.B. Silk on friendships among non-human primates (Silk, 2002). In some academic and non-academic contexts “Friendship” is still the F-word, a word that many would be reluctant to use in reference to the animal world or that “*we feel compelled to cloak it in italics, as if this gives us some indemnity against charges of anthropomorphism or lack of rigor*” (Silk, 2002). However, in our opinion “friendship” appears to be the most suitable word to describe close human-pet relationships, which imply the formation of a social bond that serves analogous emotional and adaptive functions as human-human friendships. Most of the properties that a relationship should have in order to be characterized as friendship (Silk, 2002; Brent et al., 2014) are traceable in the human-pet association: intimacy, companionship, trust, loyalty, commitment, affection, acceptance, sympathy, concern for the other's welfare, as well as time spent together and maintenance of the pair bond after long separations.

Indeed, there is plenty of evidence of a distinct role of companion animals in human lives. Many owners live closely with their pets, sharing with them their domestic space and financial resources, view them as psychological-kin and equal members of the family (Serpell, 1996; Podrazik et al., 2000; Downey and Ellis, 2008; Topolski et al., 2013). Consistently, faces of human and canine “family” members (i.e., faces associated with long-term social familiarity) evoke similar brain responses, particularly in the rostroventral anterior cingulate cortex, whose activity is considered to be associated with fundamental aspects of social cognition closely related to affection and emotion (Shinozaki et al., 2007).

## Impact and future directions

The literature reviewed so far proves that attitudes toward animals, and the development of a bond with them, may, to some degree, depend on intrinsic attributes of the animal, including physical traits and aesthetic qualities. In the next two sections some of the applicative aspects of this information will be highlighted. First, human preference for animals with specific physical characteristics will be briefly discussed taking into account its implications for animal welfare and management (mainly in reference to kennel dogs). Then, the reported neuro-hormonal bases of the human-animal bond formation will be reviewed in the light of the mounting evidence that contact with animals may affect human health and wellbeing (Friedmann and Son, 2009; McCardle et al., 2011) and considering the widespread inclusion of animals in therapeutic and educational interventions (Cirulli et al., 2011; Berry et al., 2013; Borgi et al., 2016).

### The beauty-goodness stereotype from the animal point of view

The beauty-goodness stereotype, i.e., the tendency to believe that “what is beautiful is good,” is well known in social psychology (Dion et al., 1972). People tend to believe that a person's beauty is positively related to his/her social and intellectual competence and general “goodness” (Eagly et al., 1991). Neotenous facial proportions, considered to be a component of (mainly female) facial attractiveness (Jones et al., 1995), similarly influence interpersonal impressions. Individuals with babyish faces are perceived to have childlike personality and behavioral traits, namely to be less dominant and more warm, socially dependent, physically weak, and honest than their peers with mature faces (Zebrowitz and Montepare, 1982; McArthur and Apatow, 1983; Berry, 1991).

Except for a very recent study in which dog cuteness was reported to influence strangers' ratings of dog's likely personality (namely the personality dimension of amicability, Thorn et al., 2015), the effect of the presence of infant facial features on social perception of animals has never been systematically examined. Are we victim of the beauty-goodness stereotype also when we judge animals?

The reported mix of attitudes toward dogs may represent an emblematic case. In fact, even if in several reports dogs result to be one of the most favorite species for both children and adults (e.g., Woods, 2000; Borgi and Cirulli, 2013, 2015), these animals are frequently the recipient of fear-responses from people and thus of negative attitudes (Di Nardo et al., 1988; Doogan and Thomas, 1992; Borgi and Cirulli, 2015). Previous studies have reported that dog's physical features (i.e., size, coat color and irises color, ear shape, upturn of the commissure), significantly affect human impressions and behavior (e.g., preference, tendency to approach the animal or interact/play; Wells and Hepper, 1992; Blecker et al., 2013; Fratkin and Baker, 2013; Gazzano et al., 2013; Hecht and Horowitz, 2015). There is also evidence that the difficulty with adoption for dogs kept in kennels may stem from their breed, size, age, and perceived attractiveness (Protopopova et al., 2012; Weiss et al., 2012; Svoboda and Hoffman, 2015), although further research is needed to better understand the relative importance of different factors (i.e., animal appearance and personality, cultural aspects and media influence) on dog adoption success.

In this context further analyses on human perception of cuteness appear particularly relevant, especially considering that some popular dog breeds have not a baby-like appearance (e.g., long nosed dogs such as whippets). In a recent study it has been proposed that, during domestication, dogs have evolved to manipulate the human preference for neotenous features by using the face. This study has shown that shelter dogs who exhibit facial expressions enhancing their infantile appearance (i.e., eye size and height; see also Hecht and Horowitz, 2015 for human preference for dogs with large eyes) are preferentially selected for adoption (Waller et al., 2013), a fact in line with human studies showing women's adoption preferences being dependent on cuteness perception (Volk and Quinsey, 2002).

Cuteness judgments may enhance nurturing behavior (Sherman et al., 2009; Glocker et al., 2009a) and modulate mother-infant interaction (Langlois et al., 1995). This field of analysis has the potential to be successfully translated into the human-animal interaction research, in particular by exploring to what extent animal appearance influences human-pet interaction style and care behavior toward pets. In line with human research, preliminary evidence indicate dog cuteness (at least as perceived by their owners) as one of the strongest predictor of owner-dog relationship quality, together with dog personality (a phenomenon called “Canine Cuteness Effect,” Thorn et al., 2015).

It should be taken into account that most of the research conducted so far has focused on dogs and cats (but see Kruger, 2015). Very little is known on how animal appearance influences human interactions and relationship quality with other household animals, a knowledge of increasing interest considering the popularity of small animals (e.g., rabbits, guinea pigs, or “minipigs”), including non-mammalian species, as non-traditional pets.

### Bond and benefits: animals and the “extended village effect”

There is increasing evidence that the time we invest in meaningful personal relationships serves important biological functions: the quality and quantity of face-to-face social interactions influence our immune functioning, how quickly we recover after an illness and, ultimately, how long we live (House et al., 1988; McClintock et al., 2005; Steptoe et al., 2005; Kroenke et al., 2006; Holt-Lunstad et al., 2010; Cacioppo et al., 2011). It has been called the “Village Effect,” a term which evokes a feeling of belonging to an intimate circle, “*a tight circle of people in whom you've invested serious time and affection over the years – and who have returned that attention*” (Pinker, 2014).

The question here is whether close relationships with companion animals may constitute meaningful relations as beneficial as those we establish with human friends, relatives, and romantic partners. Numerous scientific reports have shown that both long-term close relationships and short contacts with companion animals are associated with significant health effects in people, including reduced stress, lowered heart rate, and blood pressure (and thus lowered risk of cardiovascular diseases; for a review see Friedmann and Son, 2009 and McCardle et al., 2011, but see Herzog, 2011a for methodological issues in existing studies). Companion animals, especially dogs, may also indirectly benefit human health by serving as a catalyst for human-human social relationships (i.e., from incidental social interaction and getting to know people, to the formation of new friendships, Wood et al., 2015), in this way enhancing socially supportive networks.

Starting from the evidence that oxytocin and human-animal interaction effects largely overlap, it has been proposed that the activation of the oxytocin system plays a key role in the majority of the reported psychological and psychophysiological effects of human-animal interaction (Beetz et al., 2012). Coherently, direct reports of a release of oxytocin in humans in response to interaction with bonded pets are accumulating (Odendaal and Meintjes, 2003; Beetz et al., 2012), as well as evidence of oxytocin's promotion of positive social behaviors in animals toward humans (Kis et al., 2014; Romero et al., 2014).

Considering the primary role of facial cues (specifically mutual gaze) as regulating the human-dog bond mainly through oxytocin secretion (see above), a future challenge for research is to unravel the association between the strength of our attachment to pets and oxytocin-mediated health effects of human-pet interactions, and how this association is facilitated by both behavioral and facial neoteny in our ever-young companion animals. Of particular interest is the analysis of specific animal characteristics able to elicit emotional/affiliative responses in humans, especially considering its potential application to the development of interventions for social isolated subjects (Cirulli et al., 2011; Berry et al., 2013; Borgi et al., 2016). Increasing research reports indeed point in the direction of an “Extended Village Effect,” in which social connectivity with family and friends, as well as with our beloved animal friends, appears to be the best way to promote occasions for social exchange, with consequent positive effects for health and well-being.

## Conclusions

In this review we have highlighted the role of facial traits (i.e., baby schema) and facial signals (i.e., eyes gaze) as influencing human perception of animals and regulating human bond with pet species, particularly dogs.

Different studies have shown that the preference for animals with particular features (Borgi and Cirulli, 2013; Borgi et al., 2014), as well as negative attitude for some species (Borgi and Cirulli, 2015) emerge very early during development. In particular, children as young as 3 years (thus far before the reproductive age) appear to be attracted to—and to show preferential visual attention for—faces retaining infantile features (Borgi et al., 2014). This and similar evidence suggest that the presence of both physical and behavioral neotenous traits in the most common pet species might bear some part of the responsibility for our attraction to animals and motivational drive to take care of pets.

Compared to human-human communication, in human-animal interactions a more central role of facial signals can be hypothesized, mainly due to the absence of verbal language. Future research is thus needed to unravel both behavioral and neurophysiological mechanisms underlying human-animal social interaction and to what extent facial traits and facial signals may facilitate interspecific bond formation.

## Author contributions

MB drafted the manuscript; FC revised it critically for important intellectual content.

### Conflict of interest statement

The authors declare that the research was conducted in the absence of any commercial or financial relationships that could be construed as a potential conflict of interest.

## References

[B1] Ainsworth M. D. S. Bowlby J. (1991). An ethological approach to personality-development. Am. Psychol. 46, 333–341. 10.1037/0003-066X.46.4.333

[B2] Alley T. (1983). Infantile head shape as an elicitor of adult protection. Merrill. Palmer. Q. 29, 411–427.

[B3] Archer J. (1997). Why do people love their pets? Evol. Hum. Behav. 18, 237–259. 10.1016/S0162-3095(99)80001-4

[B4] Archer J. Monton S. (2011). Preferences for infant facial features in pet dogs and cats. Ethology 117, 217–226. 10.1111/j.1439-0310.2010.01863.x

[B5] Bartels A. Zeki S. (2004). The neural correlates of maternal and romantic love. Neuroimage 21, 1155–1166. 10.1016/j.neuroimage.2003.11.003 15006682

[B6] Batt S. (2009). Human attitudes towards animals in relation to species similarity to humans: a multivariate approach. Biosci. Horiz. 2, 180–190. 10.1093/biohorizons/hzp021

[B7] Beetz A. Uvnäs-Moberg K. Julius H. Kotrschal K. (2012). Psychosocial and psychophysiological effects of human-animal interactions: the possible role of oxytocin. Front. Psychol. 3:234. 10.3389/fpsyg.2012.00234 22866043 PMC3408111

[B8] Belyaev D. K. (1979). Destabilizing selection as a factor in domestication. J. Hered. 70, 301–308. 528781 10.1093/oxfordjournals.jhered.a109263

[B9] Berry A. Borgi M. Francia N. Alleva E. Cirulli F. (2013). Use of assistance and therapy dogs for children with autism spectrum disorders: a critical review of the current evidence. J. Altern. Complement. Med. 19, 73–80. 10.1089/acm.2011.0835 22978246

[B10] Berry D. (1991). Attractive faces are not all created equal: joint effects of facial babyishness and attractiveness on social perception. Pers. Soc. Psychol. Bull. 17, 523–533. 10.1177/014616729117500716866744

[B11] Bjerke T. Odegardstuen T. Kaltenborn B. (1998). Attitudes toward animals among Norwegian children and adolescents: species preferences. Anthrozoös 11, 227–235. 10.2752/089279398787000544

[B12] Bjerke T. Ostdahl T. (2004). Animal-related attitudes and activities in an urban population. Anthrozoös 17, 109–129. 10.2752/089279304786991783

[B13] Blecker D. Hiebert N. Kuhne F. (2013). Preliminary study of the impact of different dog features on humans in public. J. Vet. Behav. 8, 170–174. 10.1016/j.jveb.2012.06.005

[B14] Borgi M. Cirulli F. (2013). Children's preferences for infantile features in dogs and cats. Hum. Anim. Interact. Bull. 1, 1–15.

[B15] Borgi M. Cirulli F. (2015). Attitudes toward animals among kindergarten children: species preferences. Anthrozoös 28, 45–59. 10.2752/089279315X14129350721939

[B16] Borgi M. Cogliati-Dezza I. Brelsford V. Meints K. Cirulli F. (2014). Baby schema in human and animal faces induces cuteness perception and gaze allocation in children. Front. Psychol. 5:411. 10.3389/fpsyg.2014.00411 24847305 PMC4019884

[B17] Borgi M. Loliva D. Cerino S. Chiarotti F. Venerosi A. Bramini M.. (2016). Effectiveness of a standardized equine-assisted therapy program for children with autism spectrum disorder. J. Autism. Dev. Disord. 46, 1–9. 10.1007/s10803-015-2530-6 26210515

[B18] Bowlby J. (ed.). (1969). Attachement and Loss. London: Hogarth Press.

[B19] Brent L. J. Chang S. W. Gariepy J. F. Platt M. L. (2014). The neuroethology of friendship. Ann. N.Y. Acad. Sci. 1316, 1–17. 10.1111/nyas.12315 24329760 PMC4045505

[B20] Brosch T. Sander D. Scherer K. R. (2007). That baby caught my eye…attention capture by infant faces. Emotion 7, 685–689. 10.1037/1528-3542.7.3.685 17683225

[B21] Burnham D. Kitamura C. Vollmer-Conna U. (2002). What's new, pussycat? On talking to babies and animals. Science 296, 1435. 10.1126/science.1069587 12029126

[B22] Cacioppo J. T. Hawkley L. C. Norman G. J. Berntson G. G. (2011). Social isolation. Ann. N.Y. Acad. Sci. 1231, 17–22. 10.1111/j.1749-6632.2011.06028.x 21651565 PMC3166409

[B23] Caria A. Falco S. Venuti P. Lee S. Esposito G. Rigo P.. (2012). Species-specific response to human infant faces in the premotor cortex. Neuroimage 60, 884–893. 10.1016/j.neuroimage.2011.12.068 22230948 PMC3557818

[B24] Celani G. (2002). Human beings, animals and inanimate objects: what do people with autism like? Autism 6, 93–102. 10.1177/1362361302006001007 11918112

[B25] Cirulli F. Borgi M. Berry A. Francia N. Alleva E. (2011). Animal-assisted interventions as innovative tools for mental health. Ann. Ist. Super. Sanita 47, 341–348. 10.4415/ANN_11_04_04 22194067

[B26] De Dreu C. K. Greer L. L. Handgraaf M. J. Shalvi S. Van Kleef G. A. Baas M.. (2010). The neuropeptide oxytocin regulates parochial altruism in intergroup conflict among humans. Science 328, 1408–1411. 10.1126/science.1189047 20538951

[B27] DeLoache J. Pickard M. Lobue V. (2011). How very young children think about animals, in How Animals Affect Us: Examining the Influences of Human-Animal Interaction on Child Development and Human Health, eds McCardle P. McCune S. Griffin J. Maholmes V. (Washington, DC: American Psychological Association), 85–99.

[B28] Di Nardo P. A. Guzy L. T. Jenkins J. A. Bak R. M. Tomasi S. F. Copland M. (1988). Etiology and maintenance of dog fears. Behav. Res. Ther. 26, 241–244. 10.1016/0005-7967(88)90005-8 3408458

[B29] Dion K. Berscheid E. Walster E. (1972). What is beautiful is good. J. Pers. Soc. Psychol. 24, 285–290. 10.1037/h0033731 4655540

[B30] Doogan S. Thomas G. V. (1992). Origins of fear of dogs in adults and children: the role of conditioning processes and prior familiarity with dogs. Behav. Res. Ther. 30, 387–394. 10.1016/0005-7967(92)90050-Q 1616473

[B31] Downey H. Ellis S. (2008). Tails of animal attraction: incorporating the feline into the family. J. Bus. Res. 61, 434–441. 10.1016/j.jbusres.2007.07.015

[B32] Eagly A. Ashmore R. Makhijani M. Longo L. (1991). What is beautiful is good, but…: a meta-analytic review of research on the physical attractiveness stereotype. Psychol. Bull. 110, 109–128. 10.1037/0033-2909.110.1.109

[B33] Elia I. E. (2013). A foxy view of human beauty: implications of the farm fox experiment for understanding the origins of structural and experiential aspects of facial attractiveness. Q. Rev. Biol. 88, 163–183. 10.1086/671486 24053070

[B34] Epley N. Akalis S. Waytz A. Cacioppo J. T. (2008). Creating social connection through inferential reproduction: loneliness and perceived agency in gadgets, gods, and greyhounds. Psychol. Sci. 19, 114–120. 10.1111/j.1467-9280.2008.02056.x 18271858

[B35] Esposito G. Nakazawa J. Ogawa S. Stival R. Kawashima A. Putnick D. L.. (2014). Baby, you light-up my face: culture-general physiological responses to infants and culture-specific cognitive judgements of adults. PLoS ONE 9:e106705. 10.1371/journal.pone.0106705 25353362 PMC4212966

[B36] Farran D. C. Kasari C. (1990). A longitudinal analysis of the development of synchrony in mutual gaze in mother-child dyads. J. Appl. Dev. Psychol. 11, 419e430. 10.1016/0193-3973(90)90018-F

[B37] Feldman R. Weller A. Zagoory-Sharon O. Levine A. (2007). Evidence for a neuroendocrinological foundation of human affiliation: plasma oxytocin levels across pregnancy and the postpartum period predict mother-infant bonding. Psychol. Sci. 18, 965–970. 10.1111/j.1467-9280.2007.02010.x 17958710

[B38] Frank H. Frank M. (1982). On the effects of domestication on canine social development and behavior. Appl. Anim. Behav. Sci. 8, 507–525. 10.1016/0304-3762(82)90215-2

[B39] Fratkin J. L. Baker S. C. (2013). The role of coat color and ear shape on the perception of personality in dogs. Anthrozooös 26, 125–133. 10.2752/175303713X13534238631632

[B40] Friedmann E. Son H. (2009). The human-companion animal bond: how humans benefit. Vet. Clin. North. Am. Small. Anim. Pract. 39, 293–326. 10.1016/j.cvsm.2008.10.015 19185195

[B41] Fullard W. Reiling A. (1976). An investigation of Lorenz's “babyness”. Child. Dev. 47, 1191–1193. 10.2307/1128462

[B42] Gacsi M. Maros K. Sernkvist S. Farago T. Miklosi A. (2013). Human analogue safe haven effect of the owner: behavioural and heart rate response to stressful social stimuli in dogs. PLoS ONE 8:e58475. 10.1371/journal.pone.0058475 23469283 PMC3587610

[B43] Gazzano A. Zilocchi M. Massoni E. Mariti C. (2013). Dog features strongly affect people's feelings and behavior towards them. J. Vet. Behav. 8, 213–220. 10.1016/j.jveb.2012.10.005

[B44] Glocker M. L. Langleben D. D. Ruparel K. Loughead J. W. Gur R. C. Sachser N. (2009a). Baby schema in infant faces induces cuteness perception and motivation for caretaking in adults. Ethology 115, 257–263. 10.1111/j.1439-0310.2008.01603 22267884 PMC3260535

[B45] Glocker M. L. Langleben D. D. Ruparel K. Loughead J. W. Valdez J. N. Griffin M. D.. (2009b). Baby schema modulates the brain reward system in nulliparous women. Proc. Natl. Acad. Sci. U.S.A. 106, 9115–9119. 10.1073/pnas.0811620106 19451625 PMC2690007

[B46] Golle J. Lisibach S. Mast F. W. Lobmaier J. S. (2013). Sweet puppies and cute babies: perceptual adaptation to babyfacedness transfers across species. PLoS ONE 8:e58248. 10.1371/journal.pone.0058248 23516453 PMC3596402

[B47] Gould S. J. (1979). Mickey Mouse meets Konrad Lorenz. Nat. Hist. 88, 30–36.

[B48] Grandgeorge M. Bourreau Y. Alavi Z. Lemonnier E. Tordjman S. Deleau M.. (2015). Interest towards human, animal and object in children with autism spectrum disorders: an ethological approach at home. Eur. Child. Adolesc. Psychiatry 24, 83–93. 10.1007/s00787-014-0528-9 24590630

[B49] Gunnthorsdottir A. (2001). Physical attractiveness of an animal species as a decision factor for its preservation. Anthrozoös 14, 204–215. 10.2752/089279301786999355

[B50] Hare B. Plyusnina I. Ignacio N. Schepina O. Stepika A. Wrangham R.. (2005). Social cognitive evolution in captive foxes is a correlated by-product of experimental domestication. Curr. Biol. 15, 226–230. 10.1016/j.cub.2005.01.040 15694305

[B51] Hare B. Tomasello M. (2005). Human-like social skills in dogs? Trends. Cogn. Sci. 9, 439–444. 10.1016/j.tics.2005.07.003 16061417

[B52] Hecht J. Horowitz A. (2015). Seeing dogs: human preferences for dog physical attributes. Anthrozoös 28, 153–163. 10.2752/089279315X14129350722217

[B53] Herzog H. (2011a). The impact of pets on human health and psychological well-being. Fact, fiction, or hypothesis? Curr. Dir. Psychol. Sci. 20, 236–239. 10.1177/096372141141522026929201

[B54] Herzog H. (2011b). Some We Love, Some We Hate, Some We Eat: Why It's so Hard to Think Straight about Animals. New York, NY: Harper Perennial.

[B55] Hinde R. A. Barden L. A. (1985). The evolution of the teddy bear. Anim. Behav. 33, 1371–1373. 10.1016/S0003-3472(85)80205-0

[B56] Holt-Lunstad J. Smith T. B. Layton J. B. (2010). Social relationships and mortality risk: a meta-analytic review. PLoS Med 7:e1000316. 10.1371/journal.pmed.1000316 20668659 PMC2910600

[B57] Horn L. Huber L. Range F. (2013). The importance of the secure base effect for domestic dogs - evidence from a manipulative problem-solving task. PLoS ONE 8:e65296. 10.1371/journal.pone.0065296 23734243 PMC3667003

[B58] House J. S. Landis K. R. Umberson D. (1988). Social relationships and health. Science 241, 540–545. 10.1126/science.3399889 3399889

[B59] Hurlemann R. Scheele D. (2016). Dissecting the role of oxytocin in the formation and loss of social relationships. Biol. Psychiatry 79, 185–193. 10.1016/j.biopsych.2015.05.013 26122876

[B60] Jones D. Brace C. Jankowiak W. Laland K. Musselman L. (1995). Sexual selection, physical attractiveness, and facial neoteny: cross- cultural evidence and implication. Curr. Anthropol. 36, 725–736. 10.1086/204427

[B61] Kellert S. (1993a). The biological basis for human values of nature, in The Biophilia Hypothesis, eds Kellert S. Wilson E. (Washington, DC: Island Press), 42–69.

[B62] Kellert S. (1993b). Values and perceptions of invertebrates. Conserv. Biol. 7, 845–855.

[B63] King T. Marston L. C. Bennett P. C. (2012). Breeding dogs for beauty and behaviour: why scientists need to do more to develop valid and reliable behaviour assessments for dogs kept as companions. Appl. Anim. Behav. Sci. 137, 1–12. 10.1016/j.applanim.2011.11.016

[B64] Kis A. Bence M. Lakatos G. Pergel E. Turcsan B. Pluijmakers J.. (2014). Oxytocin receptor gene polymorphisms are associated with human directed social behavior in dogs (*Canis familiaris*). PLoS ONE 9:e83993. 10.1371/journal.pone.0083993 24454713 PMC3893090

[B65] Knight A. (2008). “Bats, snakes and spiders, Oh my!” How aesthetic and negativistic attitudes, and other concepts predict support for species protection. J. Environ. Psychol. 28, 94–103. 10.1016/j.jenvp.2007.10.001

[B66] Kringelbach M. L. Lehtonen A. Squire S. Harvey A. G. Craske M. G. Holliday I. E.. (2008). A specific and rapid neural signature for parental instinct. PLoS ONE 3:e1664. 10.1371/journal.pone.0001664 18301742 PMC2244707

[B67] Kroenke C. H. Kubzansky L. D. Schernhammer E. S. Holmes M. D. Kawachi I. (2006). Social networks, social support, and survival after breast cancer diagnosis. J. Clin. Oncol. 24, 1105–1111. 10.1200/JCO.2005.04.2846 16505430

[B68] Kruger D. J. (2015). Non mammalian infants requiring parental care elicit greater human caregiving reactions than super precocial infants do. Ethology 121, 1–6. 10.1111/eth.12391

[B69] Langlois J. Ritter J. Casey R. Sawin D. (1995). Infant attractiveness predicts maternal behaviors and attitudes. Dev. Psychol. 31, 464—472. 10.1037/0012-1649.31.3.464

[B70] Lehmann V. Huis in't Veld E. M. Vingerhoets A. J. (2013). The human and animal baby schema effect: correlates of individual differences. Behav. Process. 94, 99–108. 10.1016/j.beproc.2013.01.001 23353724

[B71] Lenzi D. Trentini C. Tambelli R. Pantano P. (2015). Neural basis of attachment-caregiving systems interaction: insights from neuroimaging studies. Front. Psychol. 6:1241. 10.3389/fpsyg.2015.01241 26379578 PMC4547017

[B72] Lišková S. Frynta D. (2013). What determines bird beauty in human eyes? Anthrozoös 26, 27–41. 10.2752/175303713X13534238631399

[B73] Little A. (2012). Manipulation of infant-like traits affects perceived cuteness of infant, adult and cat faces. Ethology 118, 775–782. 10.1111/j.1439-0310.2012.02068.x

[B74] Lobmaier J. Sprengelmeyer R. Wiffen B. Perrett D. (2010). Female and male responses to cuteness, age and emotion in infant faces. Evol. Hum. Behav. 31, 16–21. 10.1016/j.evolhumbehav.2009.05.004

[B75] Lobue V. Bloom Pickard M. Sherman K. Axford C. DeLoache J. S. (2013). Young children's interest in live animals. Br. J. Dev. Psychol. 31, 57–69. 10.1111/j.2044-835X.2012.02078.x 23331106

[B76] Lorenz K. (1943). Die angeborenen formen möglicher erfahrung [The innate forms of potential experience]. Z. Tierpsychol. 5, 233–519.

[B77] Luo L. Ma X. Zheng X. Zhao W. Xu L. Becker B.. (2015). Neural systems and hormones mediating attraction to infant and child faces. Front. Psychol. 6:970. 10.3389/fpsyg.2015.00970 26236256 PMC4505392

[B78] Luo L. Z. Li H. Lee K. (2011). Are children's faces really more appealing than those of adults? Testing the baby schema hypothesis beyond infancy. J. Exp. Child. Psychol. 110, 115–124. 10.1016/j.jecp.2011.04.002 21536307 PMC3105163

[B79] Maestripieri D. Pelka S. (2002). Sex differences in interest in infants across the lifespan: a biological adaptation for parenting? Hum. Nat. 13, 327–344. 10.1007/s12110-002-1018-1 26192926

[B80] Martín-López B. Montes C. Benayes J. (2007). The non-economic motives behind the willingness to pay for biodiversity conservation. Conserv. Biol. 139, 67–82. 10.1016/j.biocon.2007.06.005

[B81] McArthur L. Apatow K. (1983). Impressions of baby-faced adult. Soc. Cogn. 2, 315–342. 10.1521/soco.1984.2.4.315

[B82] McCardle P. McCune S. Griffin J. Maholmes V. (eds.). (2011). How Animals Affect us: Examining the Influences of Human–Animal Interaction on Child Development and Human Health. Washington, DC: American Psychological Association.

[B83] McClintock M. K. Conzen S. D. Gehlert S. Masi C. Olopade F. (2005). Mammary cancer and social interactions: identifying multiple environments that regulate gene expression throughout the life span. J. Gerontol. B. Psychol. Sci. Soc. Sci. 60, 32–41. Spec. No. 1. 10.1093/geronb/60.special_issue_1.32 15863708

[B84] Morey D. F. (1994). The early evolution of the domestic dog. Am. Sci. 82, 336–347.

[B85] Mormann F. Dubois J. Kornblith S. Milosavljevic M. Cerf M. Ison M.. (2011). A category-specific response to animals in the right human amygdala. Nat. Neurosci. 14, 1247–1249. 10.1038/nn.2899 21874014 PMC3505687

[B86] Muszkat M. De Mello C. B. Munoz Pde O. Lucci T. K. David V. F. Siqueira Jde O.. (2015). Face scanning in autism spectrum disorder and attention deficit/hyperactivity disorder: human versus dog face scanning. Front. Psychiatry 6:150. 10.3389/fpsyt.2015.00150 26557097 PMC4615933

[B87] Nagasawa M. Kikusui T. Onaka T. Ohta M. (2009). Dog's gaze at its owner increases owner's urinary oxytocin during social interaction. Horm. Behav. 55, 434–441. 10.1016/j.yhbeh.2008.12.002 19124024

[B88] Nagasawa M. Mitsui S. En S. Ohtani N. Ohta M. Sakuma Y.. (2015). Social evolution. Oxytocin-gaze positive loop and the coevolution of human-dog bonds. Science 348, 333–336. 10.1126/science.1261022 25883356

[B89] Nagasawa M. Okabe S. Mogi K. Kikusui T. (2012). Oxytocin and mutual communication in mother-infant bonding. Front. Hum. Neurosci. 6:31. 10.3389/fnhum.2012.00031 22375116 PMC3289392

[B90] New J. Cosmides L. Tooby J. (2007). Category-specific attention for animals reflects ancestral priorities, not expertise. Proc. Natl. Acad. Sci. U.S.A. 104, 16598–16603. 10.1073/pnas.0703913104 17909181 PMC2034212

[B91] Nittono H. Fukushima M. Yano A. Moriya H. (2012). The power of Kawaii: viewing cute images promotes a careful behavior and narrows attentional focus. PLoS ONE 7:e46362. 10.1371/journal.pone.0046362 23050022 PMC3458879

[B92] Numan M. Young L. J. (2016). Neural mechanisms of mother-infant bonding and pair bonding: similarities, differences, and broader implications. Horm. Behav. 77, 98–112. 10.1016/j.yhbeh.2015.05.015 26062432 PMC4671834

[B93] Odendaal J. S. Meintjes R. A. (2003). Neurophysiological correlates of affiliative behaviour between humans and dogs. Vet. J. 165, 296–301. 10.1016/S1090-0233(02)00237-X 12672376

[B94] O'Haire M. E. McKenzie S. J. Beck A. M. Slaughter V. (2013). Social behaviors increase in children with autism in the presence of animals compared to toys. PLoS ONE 8:e57010. 10.1371/journal.pone.0057010 23468902 PMC3584132

[B95] Payne E. Bennett P. C. McGreevy P. D. (2015). Current perspectives on attachment and bonding in the dog-human dyad. Psychol. Res. Behav. Manag. 8, 71–79. 10.2147/PRBM.S74972 25750549 PMC4348122

[B96] Pinker S. (2014). The Village Effect: Why Face-to-Face Contact Matters. London: Atlantic Books.

[B97] Plous S. (1993). Psychological mechanisms in the human use of animals. J. Soc. Issues 49, 11–52. 10.1111/j.1540-4560.1993.tb00907.x17165216

[B98] Podrazik D. Shackford S. Becker L. Heckert T. (2000). The death of a pet: implications for loss and bereavement across the lifespan. J. Pers. Interpers. Loss 5, 361–395. 10.1080/10811440008407852

[B99] Prokop P. Tolarovićová A. Camerik A. Peterková V. (2010). High school students' attitudes towards spiders: a cross-cultural comparison. Int. J. Sci. Educ. 32, 1665–1688. 10.1080/09500690903253908

[B100] Prothmann A. Ettrich C. Protthmann S. (2009). Preference for, and responsiveness to people, dogs, and objects in children with autism. Anthrozoös 22, 161–171. 10.2752/175303709X434185

[B101] Protopopova A. Gilmour A. J. Weiss R. H. Shen J. Y. Wynne C. D. L. (2012). The effects of social training and other factors on adoption success of shelter dogs. Appl. Anim. Behav. Sci. 142, 61–68. 10.1016/j.applanim.2012.09.009

[B102] Rilling J. K. Young L. J. (2014). The biology of mammalian parenting and its effect on offspring social development. Science 345, 771–776. 10.1126/science.1252723 25124431 PMC4306567

[B103] Romero T. Nagasawa M. Mogi K. Hasegawa T. Kikusui T. (2014). Oxytocin promotes social bonding in dogs. Proc. Natl. Acad. Sci. U.S.A. 111, 9085–9090. 10.1073/pnas.1322868111 24927552 PMC4078815

[B104] Sanefuji W. Ohgami H. Hashiya K. (2007). Development of preference for baby faces across species in humans (*Homo sapiens*). J. Ethol. 25, 249–254. 10.1007/s10164-006-0018-8

[B105] Senese V. P. De Falco S. Bornstein M. H. Caria A. Buffolino S. Venuti P. (2013). Human infant faces provoke implicit positive affective responses in parents and non-parents alike. PLoS ONE 8:e80379. 10.1371/journal.pone.0080379 24282537 PMC3840010

[B106] Serpell J. (1996). In The Company Of Animals: A Study Of Human-Animal Relationships. Cambridge: Cambridge University Press.

[B107] Serpell J. (2002). Anthropomorphism and anthropomorphic selection: beyond the “cute response”. Soc. Anim. 11, 83–100. 10.1163/156853003321618864

[B108] Serpell J. (2004). Factors influencing human attitudes to animals and their welfare. Anim. Welfare 13, 145–151.

[B109] Sherman G. D. Haidt J. Coan J. A. (2009). Viewing cute images increases behavioral carefulness. Emotion 9, 282–286. 10.1037/a0014904 19348541

[B110] Sherman G. Haidt J. (2011). Cuteness and disgust: the humanizing and dehumanizing effects of emotion. Emot. Rev. 3, 245–251. 10.1177/1754073911402396

[B111] Shinozaki J. Hanakawa T. Fukuyama H. (2007). Heterospecific and conspecific social cognition in the anterior cingulate cortex. Neuroreport 18, 993–997. 10.1097/WNR.0b013e3281ac2161 17558283

[B112] Silk J. (2002). Using the ‘F’-word in primatology. Behaviour 139, 421–446. 10.1163/156853902760102735

[B113] Siniscalchi M. Stipo C. Quaranta A. (2013). “Like owner, like dog”: correlation between the owner's attachment profile and the owner-dog bond. PLoS ONE 8:e78455. 10.1371/journal.pone.0078455 24205235 PMC3813613

[B114] Sprengelmeyer R. Perrett D. I. Fagan E. C. Cornwell R. E. Lobmaier J. S. Sprengelmeyer A.. (2009). The cutest little baby face: a hormonal link to sensitivity to cuteness in infant faces. Psychol. Sci. 20, 149–154. 10.1111/j.1467-9280.2009.02272.x 19175530

[B115] Steptoe A. Wardle J. Marmot M. (2005). Positive affect and health-related neuroendocrine, cardiovascular, and inflammatory processes. Proc. Natl. Acad. Sci. U.S.A. 102, 6508–6512. 10.1073/pnas.0409174102 15840727 PMC1088362

[B116] Stoeckel L. E. Palley L. S. Gollub R. L. Niemi S. M. Evins A. E. (2014). Patterns of brain activation when mothers view their own child and dog: an fMRI study. PLoS ONE 9:e107205. 10.1371/journal.pone.0107205 25279788 PMC4184794

[B117] Stokes D. (2007). Things we like: human preferences among similar organisms and implications for conservation. Hum. Ecol. 35, 361–369. 10.1007/s10745-006-9056-7

[B118] Svoboda H. Hoffman C. (2015). Investigating the role of coat colour, age, sex, and breed on outcomes for dogs at two animal shelters in the United States. Anim. Welfare 24, 497–506. 10.7120/09627286.24.4.497

[B119] Swain J. E. Lorberbaum J. P. Kose S. Strathearn L. (2007). Brain basis of early parent-infant interactions: psychology, physiology, and *in vivo* functional neuroimaging studies. J. Child. Psychol. Psychiatry 48, 262–287. 10.1111/j.1469-7610.2007.01731.x 17355399 PMC4318551

[B120] Thorn P. Howell T. J. Brown C. Bennett P. C. (2015). The canine cuteness effect: owner perceived cuteness as a predictor of human-dog relationship quality. Anthrozoös 28, 569–585. 10.1080/08927936.2015.1069992

[B121] Tisdell C. Wilson C. Swarna Nantha H. (2006). Public choice of species for the ‘Ark’: phylogenetic similarity and preferred wildlife species for survival. J. Nat. Conser. 14, 97–105. 10.1016/j.jnc.2005.11.001

[B122] Topál J. Gácsi M. Miklósi A. Virányi Z. Kubinyi E. Csányi V. (2005). ttachment to humans: a comparative study on hand-reared wolves and differently socialized dog puppies. Anim. Behav. 70, 1367–1375. 10.1016/j.anbehav.2005.03.025

[B123] Topolski R. Weaver N. Martin Z. McCoy J. (2013). Choosing between the emotional dog and the rational pal: a moral dilemma with a tail. Anthrozoös 26, 253–263. 10.2752/175303713X13636846944321

[B124] Trut L. N. (1999). Early canid domestication: the farm-fox experiment. Am. Sci. 87, 160–169. 10.1511/1999.2.160

[B125] Trut L. Oskina I. Kharlamova A. (2009). Animal evolution during domestication: the domesticated fox as a model. Bioessays 31, 349–360. 10.1002/bies.200800070 19260016 PMC2763232

[B126] Urquiza-Haas E. Kotrschal K. (2015). The mind behind anthropomorphic thinking: attribution of mental states to other species. Anim. Behav. 109, 167–176. 10.1016/j.anbehav.2015.08.011

[B127] Volk A. Quinsey V. (2002). The influence of infant facial cues on adoption preferences. Hum. Nat. 13, 437–455. 10.1007/s12110-002-1002-9 26193089

[B128] Waller B. M. Peirce K. Caeiro C. C. Scheider L. Burrows A. M. McCune S.. (2013). Paedomorphic facial expressions give dogs a selective advantage. PLoS ONE 8:e82686. 10.1371/journal.pone.0082686 24386109 PMC3873274

[B129] Weiss E. Miller K. Mohan-Gibbons H. Vela C. (2012). Why did you choose this pet? Adopters and pet selection preferences in five animal shelters in the United States. Animals 2, 144–159. 10.3390/ani2020144 26486914 PMC4494324

[B130] Wells D. Hepper P. (1992). The behaviour of dogs in a rescue shelter. Anim. Welfare 1, 171–186.

[B131] Wilson E. (1984). Biophilia: The Human Bond with Other Species. Harvard, MA: Harvard University Press.

[B132] Wood L. Martin K. Christian H. Nathan A. Lauritsen C. Houghton S.. (2015). The pet factor–companion animals as a conduit for getting to know people, friendship formation and social support. PLoS ONE 10:e0122085. 10.1371/journal.pone.0122085 25924013 PMC4414420

[B133] Woods B. (2000). Beauty and the beast: preferences for animals in Australia. J. Tour. Stud. 11, 25–35.

[B134] Zebrowitz L. Montepare J. (1982). Impressions of babyfaced individuals across the life span. Dev. Psychol. 28, 1143–1152. 10.1037/0012-1649.28.6.1143

